# Neurodegenerative and functional signatures of the cerebellar cortex in m.3243A > G patients

**DOI:** 10.1093/braincomms/fcac024

**Published:** 2022-02-03

**Authors:** Roy A. M. Haast, Irenaeus F. M. De Coo, Dimo Ivanov, Ali R. Khan, Jacobus F. A. Jansen, Hubert J. M. Smeets, Kâmil Uludağ

**Affiliations:** Centre for Functional and Metabolic Mapping, Robarts Research Institute, Western University, London, ON, Canada, N6A 5B7; Department of Toxicogenomics, Unit Clinical Genomics, Maastricht University, MHeNs School for Mental Health and Neuroscience, Maastricht, the Netherlands; Department of Cognitive Neuroscience, Faculty of Psychology and Neuroscience, Maastricht University, PO Box 616, 6200 MD, Maastricht, the Netherlands; Centre for Functional and Metabolic Mapping, Robarts Research Institute, Western University, London, ON, Canada, N6A 5B7; Brain and Mind Institute, Western University, London, ON, Canada, N6A 3K7; Department of Medical Biophysics, Schulich School of Medicine and Dentistry, Western University, London, ON, Canada, N6A 5B7; Department of Radiology & Nuclear Medicine, Maastricht University Medical Center, Maastricht, the Netherlands; Department of Electrical Engineering, Eindhoven University of Technology, Eindhoven, the Netherlands; School for Mental Health & Neuroscience, Maastricht University, Maastricht, the Netherlands; Department of Toxicogenomics, Unit Clinical Genomics, Maastricht University, MHeNs School for Mental Health and Neuroscience, Maastricht, the Netherlands; School for Mental Health & Neuroscience, Maastricht University, Maastricht, the Netherlands; IBS Center for Neuroscience Imaging Research, Sungkyunkwan University, Seobu-ro, 2066, Jangan-gu, Suwon, South Korea; Department of Biomedical Engineering, N Center, Sungkyunkwan University, Seobu-ro, 2066, Jangan-gu, Suwon, South Korea; Techna Institute and Koerner Scientist in MR Imaging, University Health Network, Toronto, ON, Canada, M5G 1L5

**Keywords:** m.3243A > G, cerebellum, function, structure, MRI

## Abstract

Mutations of the mitochondrial DNA are an important cause of inherited diseases that can severely affect the tissue’s homeostasis and integrity. The m.3243A > G mutation is the most commonly observed across mitochondrial disorders and is linked to multisystemic complications, including cognitive deficits. In line with *in vitro* experiments demonstrating the m.3243A > G’s negative impact on neuronal energy production and integrity, m.3243A > G patients show cerebral grey matter tissue changes. However, its impact on the most neuron dense, and therefore energy-consuming brain structure—the cerebellum—remains elusive. In this work, we used high-resolution structural and functional data acquired using 7 T MRI to characterize the neurodegenerative and functional signatures of the cerebellar cortex in m.3243A > G patients. Our results reveal altered tissue integrity within distinct clusters across the cerebellar cortex, apparent by their significantly reduced volume and longitudinal relaxation rate compared with healthy controls, indicating macroscopic atrophy and microstructural pathology. Spatial characterization reveals that these changes occur especially in regions related to the frontoparietal brain network that is involved in information processing and selective attention. In addition, based on resting-state functional MRI data, these clusters exhibit reduced functional connectivity to frontal and parietal cortical regions, especially in patients characterized by (i) a severe disease phenotype and (ii) reduced information-processing speed and attention control. Combined with our previous work, these results provide insight into the neuropathological changes and a solid base to guide longitudinal studies aimed to track disease progression.

## Teaser

The m.3243A > G mutation is known to affect brain tissue integrity. In this work, Haast *et al*. identified neurodegenerative and functional changes across the cerebellum in m.3243A > G patients using high-resolution 7 T MRI data. Together, the presented results expand our understanding of the mutation’s neurological expression.

## Graphical Abstract

**Graphical Abstract:**
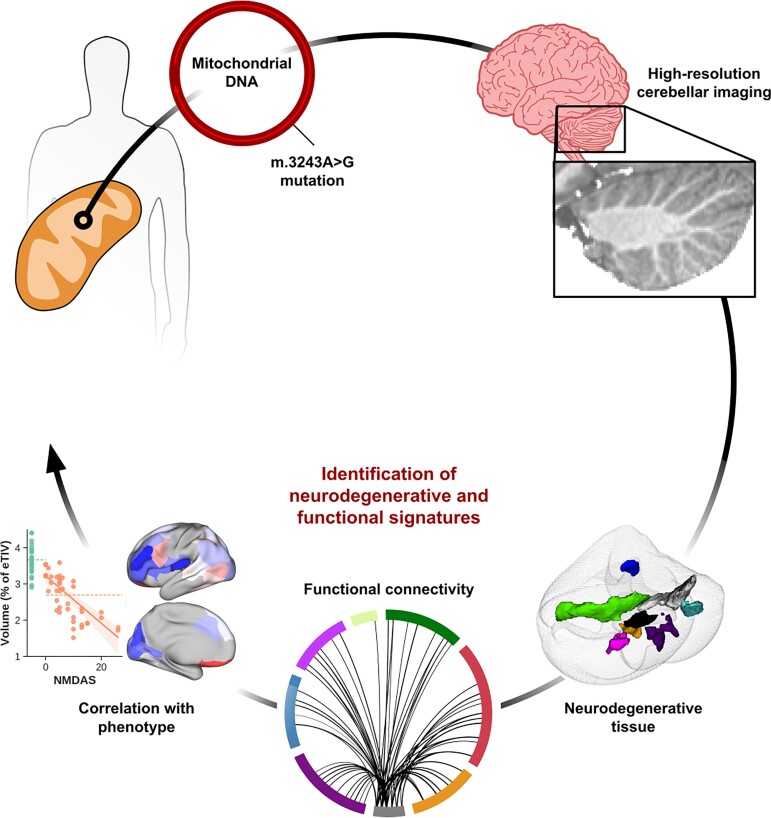

## Introduction

Among the many mitochondrial mutations reported,^1^ the adenine (A) to guanine (G) transition at base pair 3243 within the *MT-TL1* gene encoding tRNALeu (UUR), better known as the m.3243A > G mutation, has been commonly observed across the spectrum of mitochondrial disorders.^2,3^ Its clinical expression varies strongly, ranging from patients that are non-symptomatic to patients suffering from episodes of severe stroke-like symptoms.^4^ The most prominent symptoms are hearing loss (48%), gastro-intestinal symptoms (42%), decreased vision (42%), exercise intolerance (38%), glucose intolerance (37%), gait instability (36%), cerebellar ataxia (35%), myopathy (34%), cognition impairment (32%) and ptosis (32%).^5^ In symptomatic patients, the collection of symptoms are often incorrectly referred to as the ‘mitochondrial encephalopathy lactic acidosis and stroke-like episodes (MELAS)’,^6^ as stroke-like episodes are only present in 4% of the patients,^7^ or ‘maternally inherited diabetes and deafness’^8^ syndrome. Despite its relatively high prevalence compared with other mitochondrial mutations, descriptions of neuroradiological changes in m.3243A > G patients are predominantly based on single-case neuroimaging studies and only a limited number of studies have focused on larger cohorts.^9–15^

We have previously reported on the structural changes across the cerebral cortex and subcortical nuclei in a relatively large cohort of 22 m.3243A > G patients using high resolution, quantitative 7 T MRI data.^16^ We found significant volume, microstructural and perfusion differences in the brains of patients compared with healthy controls and showed that the magnitude of cerebral grey matter (GM) changes with the percentage affected mitochondria per cell (i.e. ‘mutation load’ or ‘heteroplasmy rate’) and disease severity. Here, specific cortical regions, linked to attentional control (e.g. middle frontal gyrus), the sensorimotor network (e.g. banks of central sulcus) and the default-mode network (e.g. precuneus) were shown more prone for affected tissue integrity.

Despite the sparse, but growing knowledge about the impact on the cerebral cortex, the neuroradiological correlates of the cerebellum of the m3243A > G mutation continue to remain understudied. Earlier *ex vivo* work has revealed a wide range of neuropathological findings in cerebellar tissue taken from m.3243A > G patients.^17^ Given the crucial role of mitochondria energy production in neuronal survival,^18^ a detailed in vivo characterization of cerebellar tissue changes may provide complementary insight in the neuropathological expression of the m.3243A > G mutation and its effect on overall brain’s functioning. The cerebellum features the most strongly convoluted GM across the entire human brain with densely packed neurons that together account for 78% of the brain’s entire surface area.^19^ Traditionally, it is linked to sensorimotor control, ensuring coordinated and timed movements,^20^ but its prominence across a broader range of cognitive processes has recently been confirmed through the characterization of its functional topography.^21^ Here, distinct regions within the cerebellar GM are involved in a diverse set of motor, cognitive and social and affective tasks and confirm earlier initial findings.^22–24^ As such, impaired cerebellar connectivity due to disease may have profound implications for the integrity of motor and non-motor brain networks.^25^

In this study, we extend our initial cerebral work with previously unexplored high-resolution functional 7 T MRI data to characterize (i) macroscopic and microstructural changes in the cerebellum of m.3243A > G patients and explore their (ii) spatial correspondence with the cerebellar’s anatomical and functional parcellation, (iii) effect on functional cerebello–cortical connectivity and (iv) correlation with disease severity and cognitive outcome measures. The presented results demonstrate a first and unique description of the neurodegenerative and functional signatures of the cerebellum related to the m.3243A > G mutation.

## Materials and methods

### Subject recruitment

Twenty-two m.3243A > G patients and 15 healthy controls were included in this study after providing written informed consent in accordance with the Declaration of Helsinki. The experimental procedures were approved by the ethics review board of the MUMC+ in Maastricht, the Netherlands. The participants were matched based on age, gender and education (see Table 1). A more detailed description of the in- and exclusion criteria, as well as patient characteristics can be reviewed in an earlier manuscript.^16^ Most importantly, disease-severity scores were obtained (i) by an experienced clinician (I.F.M.d.C) using the Newcastle Mitochondrial Disease Adult Scale (NMDAS, see Supplementary Table 1)^26^ and (ii) m.3243A > G mutation loads in urine epithelial cells (UECs) and blood, corrected for age and sex, respectively.^27^ Subjects in the acute phase or with a history of SLEs based on the Barthel (i.e. activities of daily living-independent) and NMDAS (<30 criteria) were excluded resulting in a spectrum with less severe phenotypes and without a diagnosis of a cerebellar motor deficit. Cognitive performance scores were collected to correlate with MRI-based findings. This included the letter-digit substitution task (LDST) to test information-processing speed, the Stroop colour-word task to test attention and the visual 15-words learning task (15-WLT) to test memory, recall and recognition.^28–30^ Raw test scores were *z*-scored based on the average control scores for each cognitive task (see Table 1). None of the patients reported subjective cognitive difficulties.

**Table 1:** Study population demographics

	Controls (*n* = 15)	m.3243A > G patients (*n* = 22)	*P-*value
Demographics			
Age, yr	38.40 (14.24)	41.23 (10.29)	0.487
Sex, % women	73.3	81.8	0.538
BMI, kg/m^2^	24.43 (4.25)	23.04 (3.60)	0.289
Education scale^a^	5.20 (1.21)	5.09 (0.92)	0.838
Disease-severity scores			
Mutation load^b^			
UECs/UECs_corrected_, %	0	53.14 (26.09)/59.77 (26.45)	—
Blood/Blood_corrected_, %	0	20.23 (11.40)/63.11 (27.38)	—
Barthel index	—	19.82 (0.83)^c^	—
NMDAS	—	8.54 (6.69)	—
Section 1—Current function	—	2.68 (3.11)	—
Section II—System-specific involvement	—	4.45 (3.63)	—
Section II—Current clinical assessment	—	1.41 (1.97)	—
Disease symptoms			
Hearing loss, % patients	—	63.6	—
Diabetes	—	59.1	—
Exercise intolerance	—	54.5	—
Tiredness	—	54.5	—
Migraine	—	36.4	—
Muscle cramps	—	27.3	—
Cardiomyopathy	—	18.2	—
Low weight	—	18.2	—
Cognitive decline	—	13.6	—
Epilepsy	—	9.1	—
Swallowing problems	—	4.5	—
Stroke-like episodes	—	4.5	—
Number of symptoms	—	3.64 (2.46)^d^	—
Cognitive performance^e^			
MMSE	29.13 (1.30)	28.27 (2.47)	0.226
LDST, *z*-score	0 (1.0)	−1.08 (2.8)	0.083
Stroop, *z*-score			
Words only	0 (1.0)	0.62 (1.35)	0.054
Colours only	0 (1.0)	0.95 (1.63)	0.081
Words and colours	0 (1.0)	1.40 (2.89)	0.127
15-WLT, *z*-score			
Total	0 (1.0)	−0.38 (1.04)	0.282
Recall	0 (1.0)	0.01 (0.99)	0.973
Recognition	0 (1.0)	−0.84 (3.01)	0.310

BMI, body mass index; UEC, urinary epithelial cells; MMSE, mini-mental state examination.

^a^ Educational scale ranges from 1 (no education) to 8 (university).

^b^ Mean heteroplasmy levels are given before and after correction for age (blood) and sex (UECs).

^c^ Maximum score is 20.

^d^ Maximum score is 12.

^e^ Significance tested using ANOVA, corrected for age, gender and education. Except for the MMSE, cognitive test scores are *z*-scored with respect to controls. *z*-Scores <0 indicate worse performance for LDST and 15-WLT, but better performance for Stroop. Values represent mean (±SD) if not stated otherwise.

### MRI acquisition

MRI data were acquired using a whole-body 7 T magnet (Siemens Healthineers, Erlangen, Germany) equipped with a 32-channel phased-array head coil (Nova Medical, Wilmington, MA, USA). High-resolution (0.7 mm isotropic nominal voxel size) whole-brain quantitative *R*_1_ and B_1_^+^ maps (2 mm isotropic nominal voxel size) were obtained using the 3D MP2RAGE^31^ and 3D Sa2RAGE^32^ sequences, respectively. *R*_1_ is an intrinsic property (i.e. longitudinal relaxation rate) of brain tissue that can be quantified using MRI and relates to tissue integrity (e.g. it decreases with demyelination).^33^ In addition to the anatomical scans, whole-brain resting-state functional MRI (rs-fMRI) data with an 1.4 mm isotropic nominal voxel size were acquired using a 2D Multi-Band Echo Planar Imaging (2D MB-EPI) sequence to probe functional connectivity between cerebellar and cortical areas. Five additional volumes were acquired with reverse-phase encoding to correct the functional data for EPI readout-related geometrical distortions. See Supplementary Table 2 for the relevant sequence parameters. Dielectric pads water were placed proximal to the temporal lobe and cerebellar areas to improve image homogeneity across the brain.^34^

### MRI data analysis

In brief, anatomical data were used to extract cerebral and cerebellar cortical GM segmentations (and surfaces) for voxel-based morphometry (VBM), while the rs-fMRI data were preprocessed to assess cerebello–cortical functional connectivity.

#### Anatomical data preprocessing

MP2RAGE anatomical data were preprocessed as described previously, including the removal of non-brain tissue,^35^ correction for image inhomogeneities^36,37^ and cortical surface reconstruction and parcellation using the FreeSurfer (v6.0).^38^ Native-resolution surface meshes (∼164 k vertices) were downsampled to the ‘fsLR’ surface space (∼32 k vertices) using the instructions and transforms provided by the Human Connectome Project (https://github.com/Washington-University/HCPpipelines).^39^

#### VBM workflow

Cerebellar neuroradiological changes in m.3243A > G patients were studied using the SUIT (v3.2, www.diedrichsenlab.org/imaging/suit.html) and VBM toolboxes in SPM12 through normalization to a spatially unbiased template of the cerebellum.^40,41^ The cerebellar GM, white matter (WM) and CSF masks were obtained using the cerebellar segmentation tool,^42^ to match the previously used labels.^16^ The sum of cerebellar GM and WM maps served as the cerebellar isolation mask and were individually checked and manually corrected using the ITK-SNAP (v3.6.0) to exclude non-cerebellar tissue.^16,43^ Diffeomorphic anatomical registration through exponentiated lie algebra^44^ was employed to normalize the individual subject’s cerebellum GM and WM masks to the corresponding probability maps of the SUIT atlas. A detailed description of the underlying workflow can be found in Diedrichsen *et al*.^45^ The resulting deformation fields were then used to deform the tissue probability and *R*_1_ maps from each individual participant. Finally, transformed GM and WM probability images were multiplied by the relative voxel volumes (i.e. the Jacobian determinants of the deformation field) to correct for volume changes during the spatial normalization step^46^ and all output was spatially smoothed with a kernel of 4 mm^3^. As a result, differences in intensities marked approximate GM or WM densities (and thus served as a proxy for tissue volume changes), and *R*_1_ for each voxel. These could then be used to directly examine differences between patients and controls (see ‘Statistical analyses’ section for further details).

### Resting-state fMRI analysis

Preprocessing of the rs-fMRI EPI volumes included slice-timing correction (using AFNI’s ‘3dTshift’),^47^ followed by estimation of (i) volume-specific motion parameter matrices (FSL’s ‘mcflirt’)^48^; (ii) gradient non-linearity (Human Connectome Project’s ‘gradient_unwarp.py’); (iii) EPI readout-related (using the opposite phase encoding images and FSL’s ‘topup’) distortions maps^49^; and (iv) the transformation to a 1.4 mm3 MNI template space. To achieve the latter, first, a linear coregistration between the subject’s mean rs-fMRI EPI volume and the subject’s native skull stripped anatomical volume (i.e. EPI-to-anatomical registration, and its inverse) was calculated using the FreeSurfer’s boundary-based registration implementation (‘bbregister’).^50^ This was followed by computing the subject’s native anatomical-to-MNI non-linear transformation warp (and its inverse) using FSL’s ‘fnirt’.^51^ Finally, each slice-timing-corrected rs-fMRI EPI volume was resampled and resliced into the MNI template space using a one-step procedure that included: (i) motion correction, (ii) gradient non-linearity, (iii) readout distortion and (iv) the MNI-space transformation.

Within the CONN functional connectivity toolbox (https://web.conn-toolbox.org/),^52^ resampled rs-fMRI data were then denoised using the aCompCor [WM and CSF region of interests (ROIs), five components each],^53^ scrubbing (number of identified invalid scans), motion regression (12 regressors: six motion parameters + six first-order temporal derivatives), temporal band-pass filtering (0.08–0.8 Hz), detrended and demeaned. In parallel, left and right hemisphere cortical (i.e. fsLR) surfaces were transformed to MNI space using the obtained inverse EPI-to-anatomical transformation matrices and Connectome Workbench’s ‘surface-apply-warpfield’ command for projection of the denoised data onto the surface.^54^

For the first-level (i.e. ROI-to-ROI) analyses, cerebello–cortical connectivity (i.e. correlation) matrices were computed for each subject. Here, ROIs included the cerebellar ROIs based on the VBM results of the anatomical data as well as predefined cortical ROIs based on the Schaefer (*n*_regions_*_ _*= 100) atlas.^55^ The Schaefer atlas exploits local gradients in resting-state functional connectivity, while maximizing similarity of rs-fMRI time courses within a parcel. It additionally allows stratification of results based on seven large-scale networks: default-mode network (DMN), frontoparietal network (FPN), dorsal attention network (DAN), ventral attention network (VAN), somatosensory motor network (SMN), limbic and visual networks.^56^

### Statistical analyses

Group and disease-severity effects were explored using the outputs from the volumetric, VBM and rs-fMRI workflows above and the statistical models implemented in the statsmodels (v1.12.0), ‘permutation analysis of linear models’^57^ and ‘network-based statistics’ (NBS)^58^ toolboxes, respectively.

Global GM, WM and lobular volumes [% of estimated total intracranial volume (eTIV) to account for differences in head size between participants] were compared between controls and patients using a one-way (GM and WM separately) or multivariate (across GM lobules) ANOVA, as well as a function of NMDAS and mutation load using linear regression analysis. Age and sex effects were accounted for by including them in the model as additional regressors.

For the VBM results and to test for between-group differences, voxel-wise comparisons were performed for GM density and *R*_1_ maps separately, after which joint inference over the two modalities was performed using the non-parametric combination (NPC) and *n *= 5000 permutations.^59^ Statistical results were corrected for age, gender and eTIV. Statistical testing was restricted to either GM or WM, as earlier results showed that the m.3243A > G genotype mostly affects GM tissue.^16^ Here, the explicit masks were obtained by thresholding (at 0.5) the corresponding SUIT cerebellar probability maps. Finally, after multiple comparison correction (i.e. across voxels and modalities)^60^ using the family-wise error (FWE, *q-FWE* = 0.05) of the statistical T-maps, corresponding clusters of significant differences were exported for visualization and used as additional ROIs for functional connectivity analyses, respectively.

Differences in ROI-to-ROI functional connectivity—defined by the Pearson’s correlation coefficients between a ROI’s across voxels averaged blood-oxygen-level-dependent (BOLD) timeseries and another ROI’s BOLD timeseries (‘edge’)—between patients and controls, were examined using the NBS statistic, while controlling for age, gender, education and eTIV. Note that the entire connectome (i.e. cortical + cerebellar ROIs) was used at this stage. Cohen’s *d* effect sizes were computed for each significant edge. Multiple regression was used to test for significant correlations of functional connectivity with disease severity and cognitive performance scores across patients only. Bonferroni correction was applied to control for multiple comparisons (i.e. *P* < 0.05/*n*_tests_).

Finally, summed ROI-based effect size maps (i.e. between groups, as well as those within patients) were decoded into a list of terms to infer mental processes from the observed pattern. To do so, the summed surface-based effect size map was projected back to volume space and smoothed using a Gaussian smoothing kernel (*σ* = 2 mm, while ignoring zero-valued voxels) using the ‘metric-to-volume-mapping’, and ‘volume-smoothing’ functions in Connectome Workbench, respectively. A GC-LDA model, in conjunction with results from 14 371 studies within the Neurosynth database, was then used to extract a set of terms. The resulting term’s weight is associated with its relative spatial correspondence with the statistical map’s cortical pattern.^61,62^

## Data availability

All automatic anatomical and functional data (pre-)processing steps as detailed above have been implemented in a custom and publicly available Snakemake^63^ workflow (https://github.com/royhaast/smk-melas). Raw and processed patient data cannot be made publicly available due to institutional privacy restrictions.

## Results

**Figure 1:**
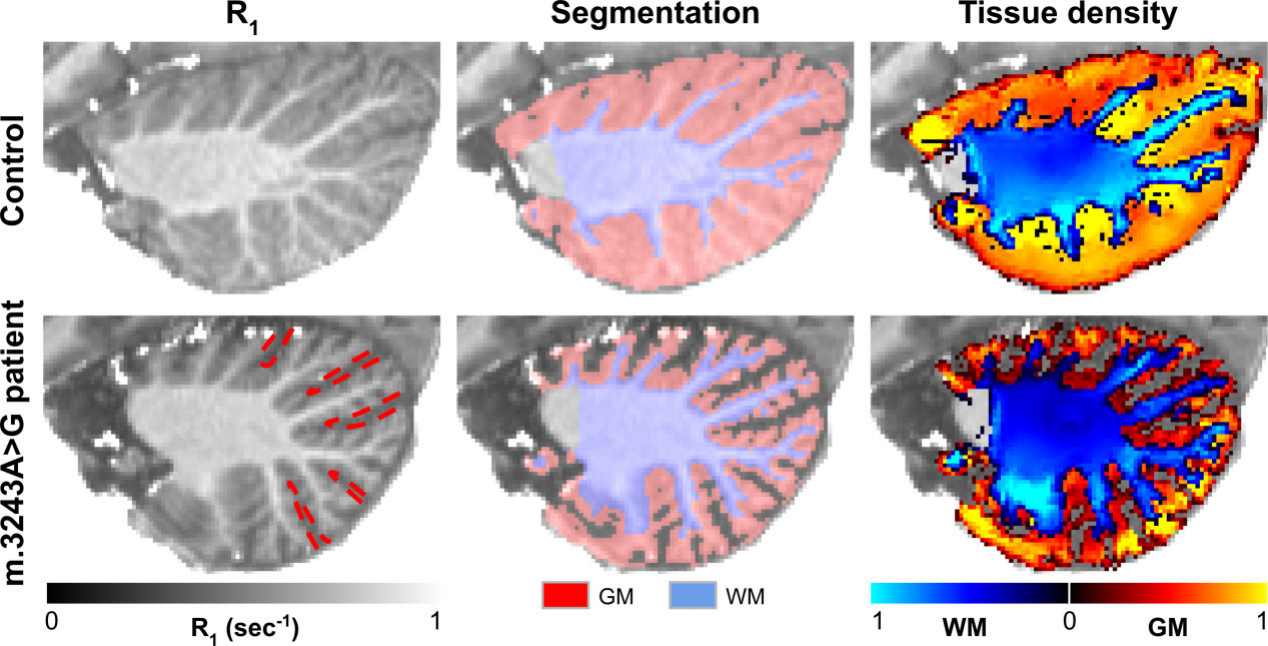
**Example data.** Left to right: *R*_1_, GM (red) and WM (blue) segmentation masks and corresponding tissue density maps are shown for a control (*top* row) and m.3243A > G patient (*bottom* row). Dashed red lines indicate the inter-folial spacing for the patient.

Example quantitative *R*_1_ (s^−1^, left column), cerebellar tissue masks (middle) and density map (a.u., right) for a control subject (top row) and an m.3243A > G patient (bottom row, 24 versus 38 years old, respectively) are depicted in Fig. 1 across a single sagittal slice. As can be observed, larger inter-folial spaces are visible in the *R*_1_ (first column) and segmentation images (middle column) for the patient, as indicated by the dashed red lines, compared with the control subject.

**Figure 2:**
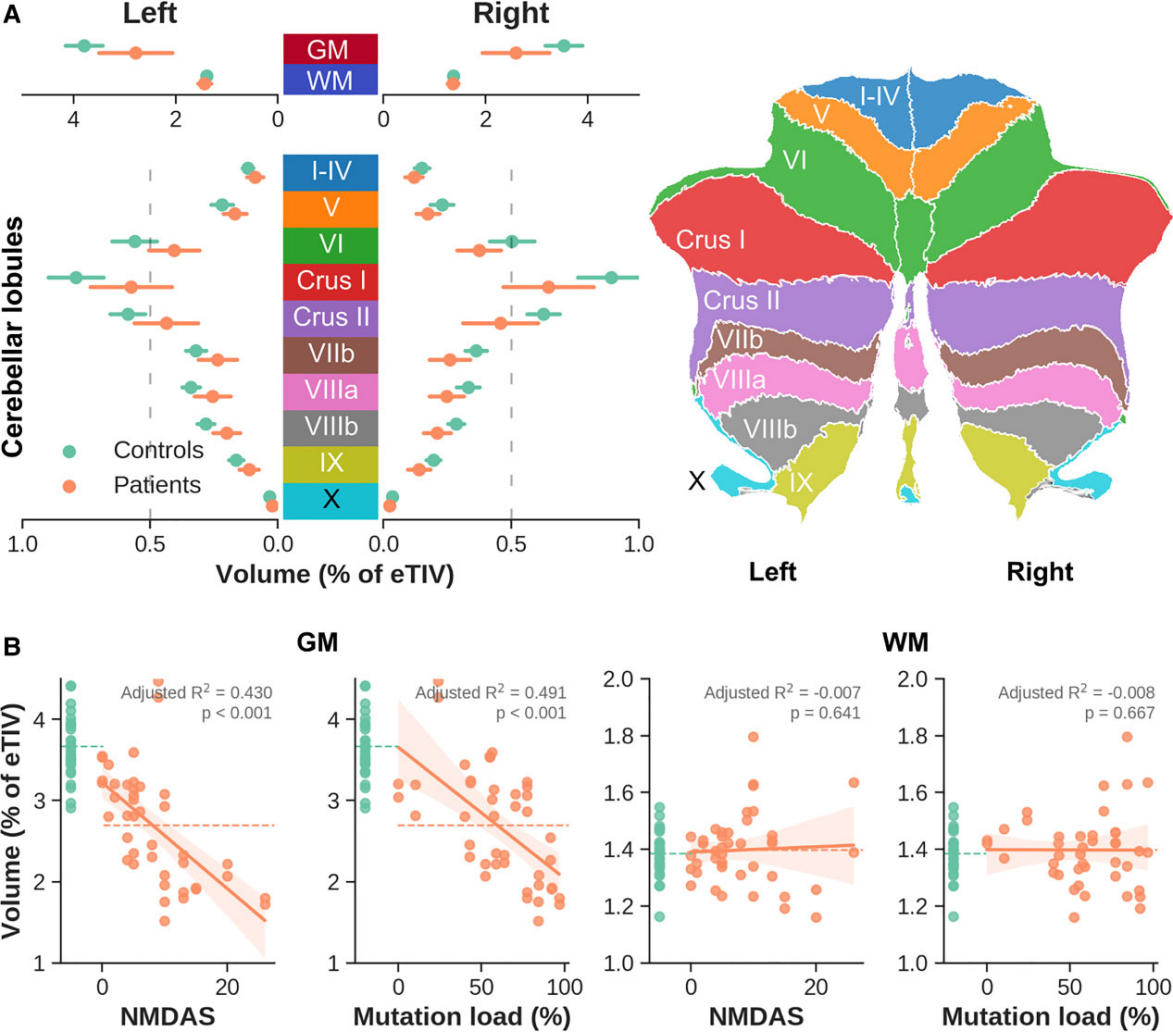
**Cerebellar GM and WM volumes.** (**A**) Comparison of volume (presented as % of eTIV on the *x*-axis) between controls (green) and m.3243A > G patients (orange) for left and right hemisphere GM and WM (*top*), as well as per cerebellar lobule GM (*bottom*), colour-coded based on the right panel legend. (**B**) First two columns: correlation between GM volume (*y*-axis) and NMDAS or corrected UEC mutation load (*x*-axes). Last two columns: similar to first two columns but using WM volume (*y*-axis). Shaded areas show 95% confidence intervals.

Average GM volume was significantly lower for the patient group [*F*(1,68) = 14.96, *P* < 0.001, corrected for age, gender and eTIV], while this main effect was negligible for WM [*F*(1,68) = 0.733, *P* > 0.05, see red versus blue dots in top panel in Fig. 2]. Significant correlations between average GM, not WM, volume and NMDAS (*P* < 0.001) and UEC mutation load (corrected for sex, *P* < 0.001) were observed. This pattern is consistent across (un)corrected heteroplasmy levels in both UEC and blood, while most apparent for the NMDAS section II subscore, and LDST and Stroop cognitive scores (see Supplementary Fig. 1).

**Figure 3:**
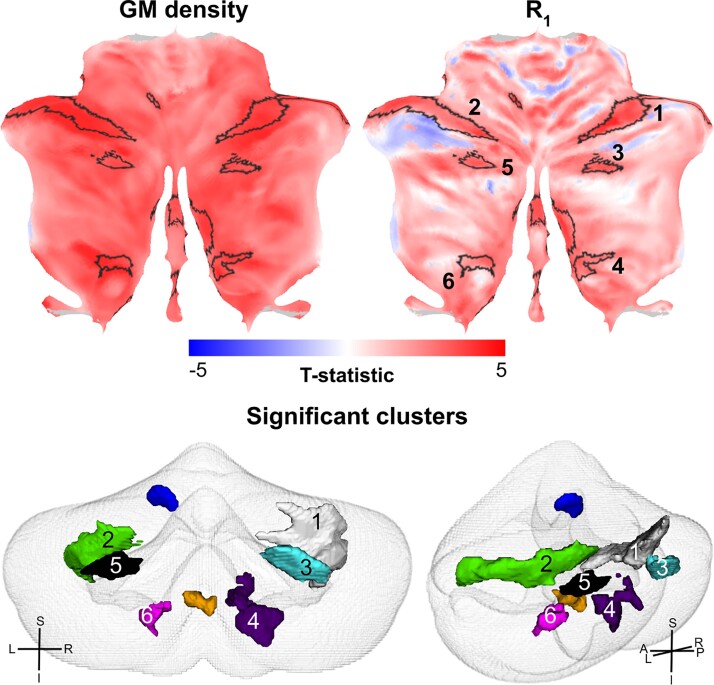
**Voxel-based statistical results.** Flatmap representation of the statistical result when comparing GM density (left) and *R*_1_ (*middle*) maps between controls and patients. Significant clusters after joint interference are delineated using solid black lines on the flatmaps and represented as 3D meshes (*bottom*). Orientation crosses provide references to left–right (L–R), superior–inferior (S–I) and anterior–posterior (A–P) axes.

More detailed, voxel-wise comparison of GM density and *R*_1_ in Fig. 3 were used to better describe the spatial-specificity of volumetric differences between groups. Both modalities were tested individually and then combined for joint inference using Fisher’s NPC to extract significant clusters. Note that results are visualized on a flat representation of the cerebellum but that the analyses were performed in volume space (see Supplementary Fig. 2 for the volume to flat representation correspondence). GM density was found consistently higher for control subjects (i.e. in red), while differences in *R*_1_ are more variable but reveal a similar pattern with higher *R*_1_ for the controls. A total of eight clusters of voxels characterized by significant differences in both GM density and *R*_1_ (Fisher combined *P*_permuted_*_ _*< 0.05, delineated by solid back lines) were extracted. The six largest clusters (1–6), characterized by a symmetric distribution across left and right hemispheres (see also 3D rendering), were selected for further characterization using public atlases as well as *in vivo* rs-fMRI data.

**Figure 4:**
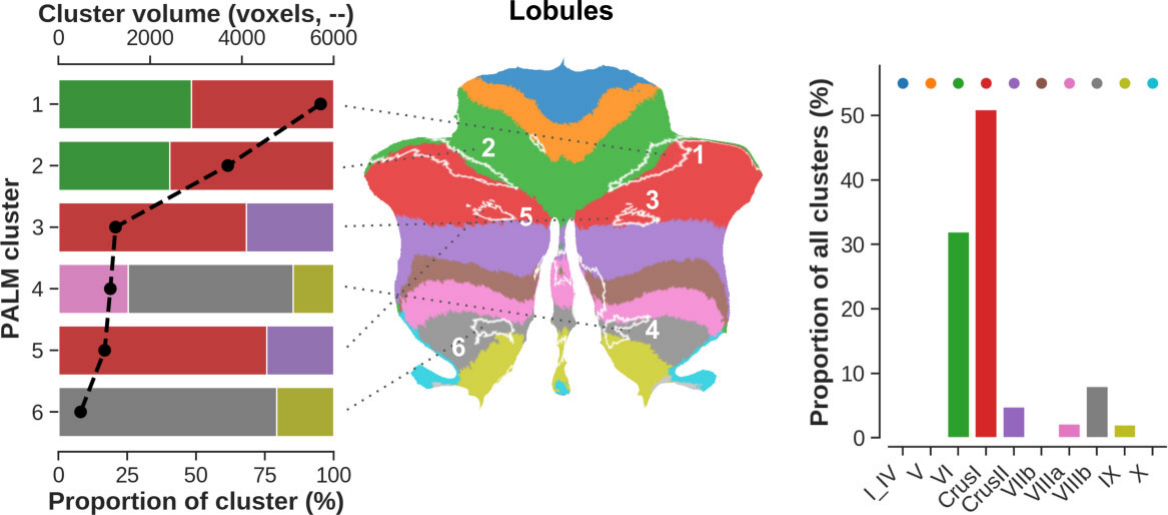
**Spatial distribution of the significant clusters with respect to the cerebellar lobules.** Left to right: stacked bar plot showing statistical (i.e. PALM) clusters (*y*-axis), ordered from largest at the *top* (Cluster 1) to smallest at the *bottom* (cluster six, in voxels, *top x*-axis). Here, the width of each individually coloured bar represents the proportional overlap (*bottom x*-axis) with the respective lobule. For example, 50% of cluster one overlaps with Crus I. *Middle* panel shows a flatmap representation to visualize the localization of each cluster across the cerebellar GM with respect to its lobules. Right panel shows the proportional overlap (*y*-axis) across all clusters per lobule (*x*-axis). For example, 50% of significant voxels fall within Crus I.

First, to evaluate whether the significant clusters tend to colocalize with predefined anatomical (or functional) parcels, we quantified cluster sizes and their overlap for each cluster–parcel combination (e.g. Cluster 1 versus Lobule VI, Fig. 4, left panel). Here, the dashed black line represents the individual cluster sizes (in number of voxels, sorted from largest to smallest) while the stacked bar plot indicates the proportion (%) of each cluster that falls within the respective colour-coded atlas region (see middle panel). The two largest clusters (i.e. one and two, covering 1960 and 1266 mm^3^, respectively) were equally positioned across Lobule VI (48.31 and 40.36% of their total volume, respectively) and Crus I (51.96 and 59.64%). Cluster sizes drop strongly from Cluster 3 with volumes decreasing from 427 to 166 mm^3^. Taken together (right panel), Lobule VI (32.01%) and Crus I (50.97%) show the largest overlap with all clusters.

Functionally (see Supplementary Fig. 3), Clusters 1 (74.36%) and 2 (72.48%) strongly colocalize with FPN. Overall, most voxels characterized by a significant difference in GM density and *R*_1_ between groups lie within FPN (52.57% of total voxels), followed by the DMN (26.21%), VAN (13.96%) and SMN (6.66%), while the overlap with visual, DAN and limbic networks remain negligible (i.e. <1%).

**Figure 5:**
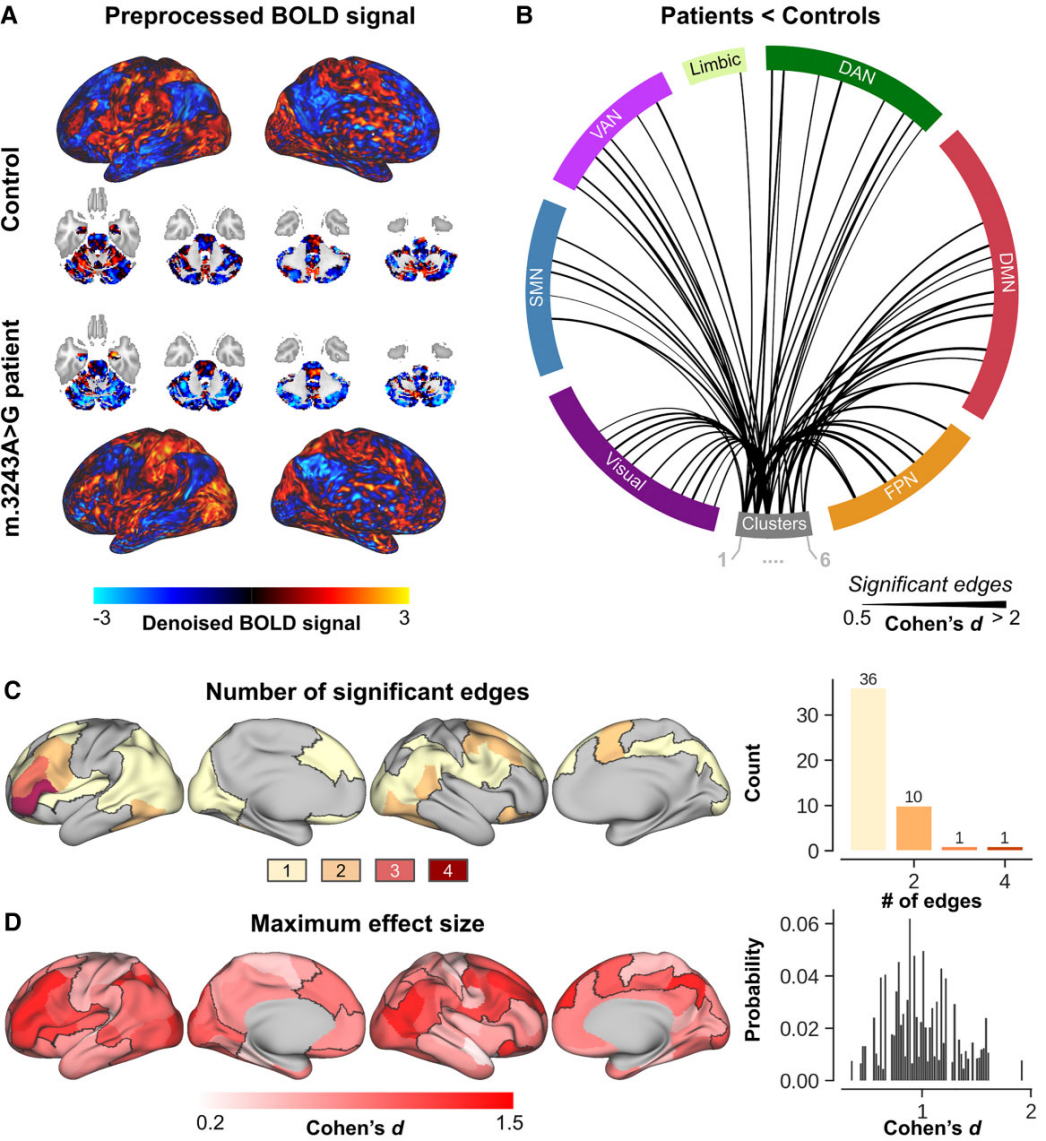
**Characterization of cerebello–cortical functional connectivity.** (**A**) Visual comparison of the denoised rs-fMRI cortical and cerebellar data for a control subject (*top* part) and m.3243A > G patient (*bottom* part) at a corresponding brain coactivation timepoint. (**B**) Significantly reduced (solid black lines) cerebello–cortical (separated per large-scale brain network) connections in m.3243A > G patients compared with controls. Significant connections were identified using the NBS statistic, while controlling for age, gender, education and eTIV. (**C**) Surface-wise visualization of the total number of significantly reduced edges (in m.3243A > G patients) per cortical ROI. For example, a cortical ROI will be coloured yellow if it shows reduced connectivity to only a single cerebellar cluster, but red if it shows reduced connectivity to four out of the six clusters. ROIs not affected at all are shown in grey. (**D**) Corresponding maximum effect size per ROI. Briefly, all ROIs are characterized by six *T*-statistical values, based on the group-wise difference for each of the cerebellar clusters. The maximum is then mapped onto the cortical surface.

Second, to characterize the functional signatures of the affected tissue, connectivity profiles (i.e. ROI–ROI functional timeseries correlation) extracted from *in vivo* rs-fMRI data were explored and compared between groups. Example rs-fMRI cortical and cerebellar data for a control subject and m.3243A > G patient for a corresponding brain coactivation timepoint (i.e. DMN) are shown in Fig. 5A. Subsequent statistical comparison between groups revealed one network across the cortical and cerebellar nodes with 167 edges that were characterized by a significant reduction in connectivity strength for the m.3243A > G patients. Supplementary Fig. 4 shows the statistical and corresponding significance matrices. Across all 167 edges, 63 edges (37.72%, solid black lines in Fig. 4B) showed a significantly impacted (*P* < 0.05, NBS corrected, controls > patients) connectivity strength between the cerebellar clusters and a cortical ROI. No difference was observed between left and right cerebral hemispheres in their connectivity to the cerebellum in patients [*F*(1,49) = 0.008, *P* > 0.05]. See Supplementary Figs. 5 and 6 for the cluster-wise Cohen’s *d* effect sizes and cortical connectivity profiles, respectively. Taken together, these affected cortical ROIs (delineated using a solid black line in Fig. 5C and D) are predominantly positioned along a lateral parietal to frontal band where most prominent group effects are observed in the (especially left hemispheric) frontal regions with cortical ROI’s characterized by reduced connectivity with at least two cerebellar clusters (Fig. 5C). In parallel, the m.3243A > G mutation most significantly impacts the frontal regions (Fig. 5D).

**Figure 6:**
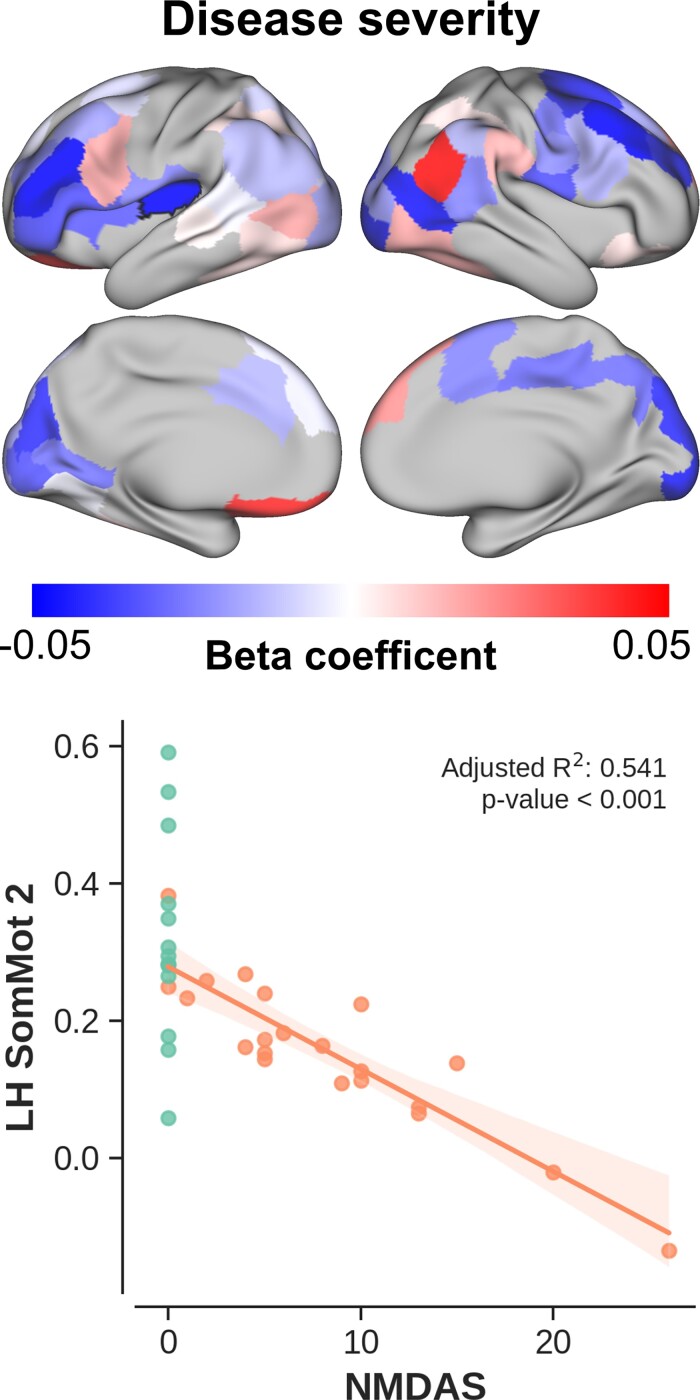
**Disease severity versus connectivity.**  *Top* panel: beta coefficients (i.e. explained change in connectivity strength per unit change in NMDAS) per cortical region mapped onto the cortical surface. *Bottom* panel: scatter plot showing the change in connectivity (for patients, in orange) as function of NMDAS for the cerebello–cortical pair characterized by the strongest correlation. Control data are shown for comparison (in green). Shaded areas show 95% confidence intervals.

**Figure 7:**
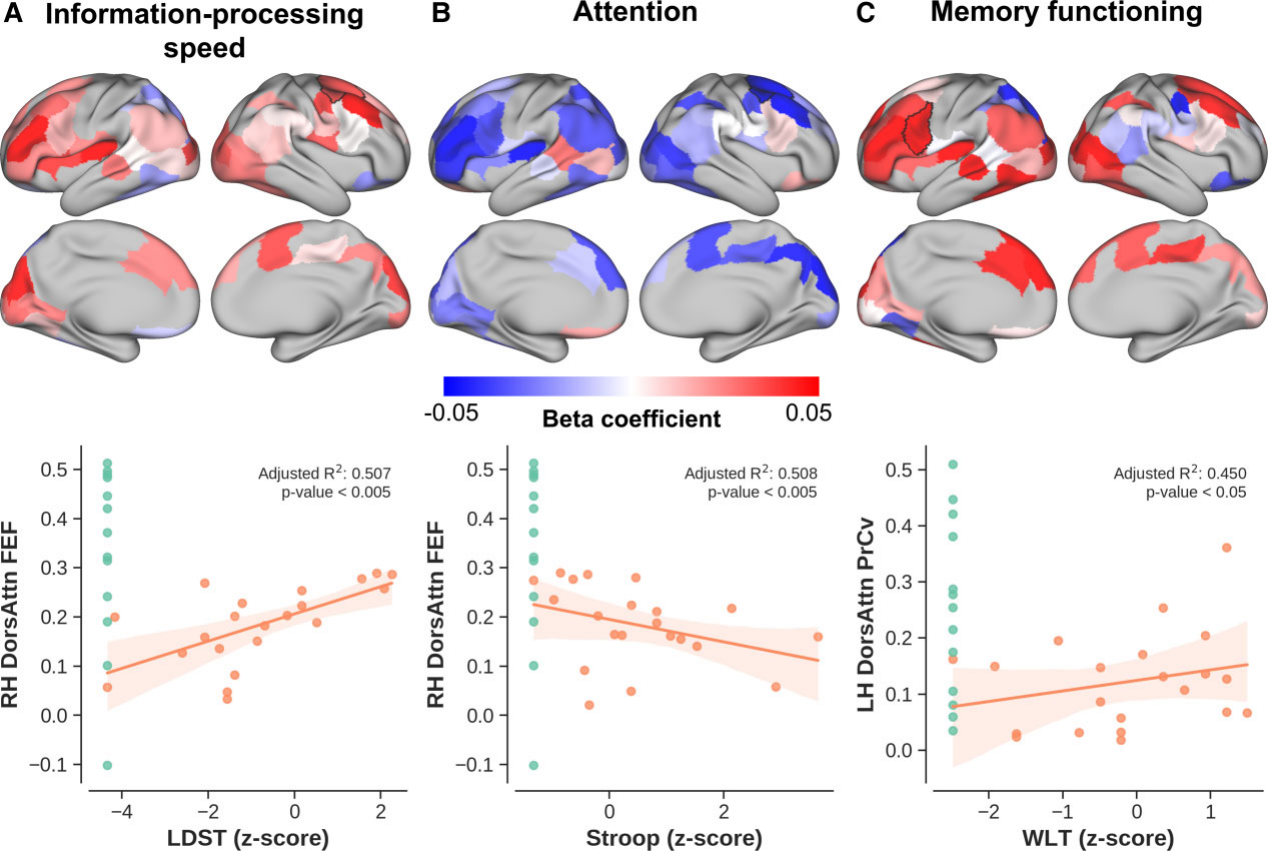
**Cognition versus connectivity.** Similarly to Fig. 6 with *top* panels showing the beta coefficients (i.e. explained change in connectivity strength per unit change in cognitive test score) per cortical region and *bottom* panels showing scatter plot with the change in connectivity as function of (**A**) LDST, (**B**) Stroop and (**C**) WLT corresponding to information-processing speed, attention and memory functioning, respectively. Shaded areas show 95% confidence intervals.

Once we identified the edges that were statistically reduced in the patient group, correlation analyses were used to investigate whether the observed effect was stronger in patients characterized by (i) a more severe disease phenotype (Fig. 6) or (ii) worse cognitive performances (Fig. 7). Overall, but not exclusively, functional connectivity scales negatively with increasing NMDAS score (i.e. more severe phenotype) across patients. Again, this effect is strongest in the frontal lobe, as well as the insular cortex. For example, a negative correlation (*P* < 0.001) is visible between NMDAS and cerebellar functional connectivity to a region embedded within the SMN (outlined with a black solid line in the upper left surface-based display). Positive correlations are observed across several regions too. However, in contrast to the negative correlations, these are spread asymmetrically across the brain, without a strong spatial preference.

Functional connectivity decreases with decrease in cognitive performance based on the patients’ LDST (i.e. higher is better, see Fig. 7A for the corresponding cortical ROI beta coefficients), Stroop (i.e. higher is worse, Fig. 7B) and 15-WLT (higher is better, Fig. 7C) test scores. This effect is most consistent across regions for LDST (i.e. information-processing speed) and Stroop (attention), but more variable for WLT (memory).

Group-wise, disease severity and cognitive performance effect sizes (see Supplementary Fig. 7A for their comparison) were summed to identify cortical regions characterized by the most consistent change in their functional connectivity with the cerebellar clusters. Summed effect sizes ranged from 4.31 in parietal regions up to 16.99 in frontal regions (Supplementary Fig. 7B). Comparison of the corresponding spatial pattern to the results extracted from 14 371 studies in the Neurosynth database revealed a strong correlation with broad terms such as ‘visual’ (‘correlation weight’ = 10,097.54, Supplementary Fig. 7C), ‘motor’ (5161.05) and ‘attention’ (3906.84) where the term’s font size scales with its corresponding weight.

## Discussion

The m.3243A > G genotype is characterized by a large phenotypic spectrum across patients.^2,4^ In this work, we employed the most detailed MRI dataset available in a relatively large population of patients carrying the m.3243A > G mutation to define alterations of the spatial pattern of cerebellar macro- and microstructural features, as well as their functional connectivity to cortical areas.

### Impact on cerebellar structure

In line with our earlier cerebral cortical findings,^16^ the current results show that the m.3243A > G mutation-induced (almost exclusively) cerebellar GM tissue changes. Cerebellar GM atrophy worsened with increased severity based on the NMDAS score as well as a higher mutation load measured in both blood and UECs similar to that observed for the cerebral cortex. This follows previous *in vivo* and *ex vivo* observations by means of a higher degree of abnormal radiotracer binding^15^ and neuronal loss^17^ in cerebellar tissue from more severely affected patients, respectively. The GM density changes were accompanied by a decrease in *R*_1_, indicating a reduced concentration of intracortical myelin and iron.^33^ In contrast, the WM tissue remained unaffected, independent of disease severity based on both clinical phenotype and mutation load. Together these suggest that the GM tissue’s integrity can become severely impaired in m.3243A > G patients, compared with a group of controls. While this effect appeared to be global (i.e. across the entire GM), statistical testing revealed several ‘hot spots’, or clusters, spread across the cerebellar lobules in a systematic left versus right fashion for the largest clusters. Spatial characterization of these clusters with respect to a cerebellar anatomical atlas and its lobulation^45^ revealed a strong bias towards lobules VI and Crus I, harbouring almost 80% of all the significant voxels. In the following, we will contextualize these results using the relevant literature, focusing mostly on the interplay between mitochondrial (dys)functioning and neuronal integrity.

As mentioned in the ‘Introduction’ section, the cerebellum is known for its immensely folded structure that accounts for the majority of the neuronal cell bodies found in the brain. It covers a total area of about 1590 cm^2^ when unfolded, rendering it considerably more dense compared with the roughly 2000 cm^2^ area of the eight times volume of the larger cerebral cortex.^19,64^ Consequently, the cerebellar tissue requires a steady and relatively vast supply of nutrients (mostly carbohydrates and fatty acids) to nourish the basal level of activity of its densely packed neurons.^65^ The metabolic processes to release the stored energy from these nutrients and generate ATP, the actual energy substrate, is coregulated by a collection of respiratory chain subunits located within the mitochondria.^66^ As such, mitochondrial mutations, like the one central to this work, will lower the mitochondria’s efficiency to produce ATP through oxidative phosphorylation^67^ and affect the functioning of multiple organs, when crossing a tissue-specific threshold.^68^ Below the threshold, the mutation remains unnoticed. It has been shown in myoblasts (i.e. embryonic progenitor cells that give rise to muscle cells) from a single MELAS patient that having a >80–90% m.3243A > G mutation load leads to impaired translation of all mitochondrial encoded respiratory chain subunits with a decrease in ATP synthesis as result.^69^ Recent work has confirmed this observation in human neurons using induced pluripotent stem cell (iPSC) technology.^70^ Additionally, the authors observed differences between low and high levels of heteroplasmy iPSC neurons’ anatomy where high levels (71%) of the m.3243A > G mutation appeared to reduce synapses, mitochondria and dendritic complexity. This is in line with earlier work that linked mitochondrial dysfunction, as well as reduced mitochondrial mass, with altered neuronal dendritic morphology and remodelling *in vitro* and *in vivo*, including direct measurements in the cerebellum.^17,70,71^ Additionally, simulations based on a m.3243A > G biophysical model suggest that cell volume decreases with increasing heteroplasmy to prevent potential energy crises^72^ while the absolute number of mitochondria is often increased in m.3243A > G patients.

Moreover, biochemical deficits and clinical implications only appear once the patient’s heteroplasmy level surpasses a certain cellular or tissue-specific threshold.^1,67,73^ As we only included patients, this implies that the threshold at least in some tissues was surpassed, although symptoms could be very subtle and with cerebellar GM volumes similar to those in the lower regime of observations across healthy controls. The linearly (and significantly) decreasing GM volume as a function of mutation load is indicative of an additional gradual effect of the genotype on the cerebellar tissue changes once the threshold of expression is surpassed (i.e. more profound enzyme deficiency). It is important to note that a similar, linear relationship was observed when opposing the volumetric measures to the NMDAS score. Patients with a more severe disease phenotype appear to be characterized by the strongest atrophy. Nevertheless, the heterogeneity and complexity of the m.3243A > G phenotype challenge theoretical understanding of their causation and require longitudinal tracking of disease progression.

Taken together, the observations discussed above strongly suggest that the m.3243A > G mutation specifically impacts the GM tissue through neuronal morphological changes. Here, our spatial characterization using voxel-wise analyses—that showed a bias towards lobules VI (∼30%) and Crus I (∼50%), located along the superior-posterior portion of the cerebellum—might be used to further deduce the anatomical specificity of these changes towards specific cytoarchitectonic, molecular and/or structural connectivity features.^74^

Cytoarchitectonically, the cerebellar GM is characterized by a distinct (i.e. compared with the neocortex), uniform three-layer architecture composed of the inner granular, outer molecular layer and in between a sheet of Purkinje cells which are solely responsible for directing information away from the cerebellum.^75^ Independent of lobulation, ‘transversal zones’ have been identified by leveraging the molecular topography defined by the expression of specific genes across the cerebellum. Interestingly, most significant voxels lie within a central zone characterized by Purkinje cells expressing zebrin II,^76^ which is analogous to aldolase C72,^77^ an important player in glycolytic ATP biosynthesis,^78^ posing an indirect link to mitochondrial dynamics.^79^ While spinocerebellar ataxia seems to involve neurodegeneration of motor-related cerebellar regions,^80^ m.3243A > G-related atrophy might be restricted to certain Purkinje subtypes (e.g. zebrin II+). However, the molecular characterization remains a complex issue and out of the scope of this manuscript. In parallel, the cerebellar cortex can be parcellated based on its anatomical connectivity. In contrast to the transversal zones based on genetic markers, these zones run in a longitudinal fashion, perpendicular to the long axis of the lobules. Most significant voxels lie within zones that appear to receive input from the principal olive nucleus. However, the current results do not show a clear bias towards a specific (set of) zone(s) with the significant clusters spanning from the lateral hemispheres up to the (para)vermis. More coarsely, tracer studies in the macaque monkey show a distinction between prefrontal (mainly lobules Crus I and II) and motor (all other) modules, with anatomical connections running to the respective cortical areas.^23,81^ With Crus I being the most affected lobule, especially prefrontal connectivity might be impacted.^82^ However, *in vivo* fMRI data are necessary to characterize the functional consequences, which will be discussed next.

### Impact on cerebellar functional connectivity

It is the growing consensus, supported by electrophysiological mapping in a range of species, that the cerebellum’s functional modules are not shaped by its lobules but extend beyond its fissures.^74^ Drawing conclusions solely based on comparisons with previously published anatomical parcellations and literature might therefore paint an incomplete picture. As such, we leveraged an openly available functional parcellation, as well as acquired rs-fMRI data to more precisely map out the impact of the observed differences on the brain’s functioning, and potential correlations with the clinical phenotype, based on disease severity and cognitive performance.

Several studies have used the synchronization of rs-fMRI signals between brain regions to identify seven large-scale brain networks.^56,83^ From a historic perspective, the function of the cerebellum has been linked to the sensorimotor system. However, the cerebellum appears to play an important role across multiple of the identified large-scale cortical brain networks.^84,85^ Our results show great overlap with cerebellar fractions of four of these identified networks but most prominently with FPN (>50%), followed by DMN (∼25%) and VAN (∼15%). All regions that show functional connectivity with associative regions of the cerebral cortex (and found to be similarly affected in schizophrenic patients).^86^ The FPN, also known as the ‘central executive network’, plays an important role in higher cognitive functions by actively maintaining and manipulating information in working memory, for rule-based problem solving and for decision making in the context of goal-directed behaviour.^87^ Unlike all other networks, the FPN is disproportionately (i.e. ∼2-fold) expanded in the cerebellum compared with the cerebral cortex and might therefore play a relatively important role at the whole-brain scale.^84,88^ Damage to the FPN in the cerebellum disturbs a broad range of control functions, including task switching, working memory retrieval, visuo-spatial integration, language and an overall reduction in intellectual function,^89^ collectively known as the cerebellar cognitive affective syndrome.^90^ Cognitive deficits are not uncommon in mitochondrial disorders and prevalent in up to a third of m.3243A > G patients.^5,68^ While cognitive performance appears to reduce in general, distinct domains, including verbal comprehension, perceptual reasoning, working memory, processing speed and memory retrieval, were found to be affected in particular.^91^ Similarly, the lower LDST and Stroop test scores indicate impaired information-processing speed and attention in the current cohort of patients. In both cases, adequate performance thrives on the fluent selection of relevant visual features through neuronal computations in frontal, parietal and/or limbic areas that are then projected to occipital (i.e. visual) areas.^92,93^

Additionally, we used rs-fMRI data to identify impaired brain networks in our patients. Prior evidence is scarce and only one study has systematically investigated changes in the whole brain’s functional topology of m.3243A > G patients.^94^ Here, modularity analysis (e.g. network efficiency) revealed that patients had altered intra- or inter-modular connections in default mode, frontoparietal, sensorimotor, visual and cerebellum networks. Our results—using analyses that were particularly focused on the interplay between the affected cerebellar clusters and the rest of the brain—revealed a single network of regions that showed significantly reduced connectivity in the m.3243A > G patients. Spatial characterization of this network shows a strong emphasis on frontal and parietal lobe regions with especially the (left) frontal lobe characterized by impaired connectivity with the cerebellum (e.g. based on the number of significant edges) that intensifies in the more severely affected patients, based on the NMDAS score. This bias towards the frontal lobe, also known as fronto-cerebellar dissociation, has been found to increase the difficulty for a person to select the appropriate response to a stimuli, or to initiate the response (i.e. executive functioning).^95^ Moreover, focal frontal and parietal lobe lesions resulted in increased errors and slowness in response speed during the Stroop test.^96,97^ Similarly, the frontal-parietal cortical network appears to be strongly engaged during the LDST task.^98^ In line with these previous studies, our correlational analyses between functional and cognitive profiles show that cerebello–cortical connections characterized by a significant group effect, are weaker in patients with lower LDST and Stroop performances. Additionally, the left frontal lobe is considered the anterior convergence zone of the dorsal (i.e. phonology) and ventral (i.e. semantics) language streams,^99^ thus playing an essential role in this dual-stream model. The central role of the frontal lobe in this model of language processing explains the appearance of terms like ‘language’, ‘words’ and ‘semantic’ when comparing our statistical maps to those included in the NeuroSynth database^62^ and could provide novel insights into the cognitive deficits related to the m.3243A > G mutation, and/or mitochondrial diseases in general.

### Clinical implications

The clinical manifestation of the m.3243A > G mutation is characterized by wide variability in nature and severity of symptoms.^4^ In a small subset of carriers, the mutation induces a severe phenotype, such as the MELAS syndrome, with stroke-like episodes, encephalopathy and progressive cognitive difficulties.^4,7^ Current results—based on mildly affected patients with relatively low Barthel and NMDAS scores—are therefore most relevant for more common manifestations (e.g. MIDD and myopathy), and patients characterized by a mutation load range like the current study population, while generalizability to more severe m.3243A > G clinical phenotypes is lower. Regardless, cerebellar integrity, in particular the subregions identified by the current work, could serve as a target for longitudinal disease tracking (e.g. to study brain–phenotype relationship) and/or evaluate the efficacy of potential treatments (e.g. l-arginine supplementation) across the entire spectrum of patients.^100^ Based on current and previous^16^ findings, structural changes in m.3243A > G patients range from large-scale deformations (e.g. enlarged ventricles) to fine-scale (e.g. local tissue *T*_1_) changes, depending on the severity of the case. While ventricular volume changes can readily be detected at conventional field strengths (i.e. ≤3 T), the use of 7 T MRI might be crucial to detect the subtle differences in structures like the cerebellum. The steady increase in the number of clinically approved 7 T MRI scanners will increase the feasibility to apply these methodologies in more clinical-oriented applications (e.g. diagnosis, drug development).

### Technical considerations

Despite the advantage of using high-resolution anatomical and functional data, the cerebellum’s fine-scale anatomy might introduce signal contamination.^19^ Partial voluming effects (in regions characterized by a thin cortex) between the GM, WM and CSF voxels’ fMRI timeseries, in particular, will affect downstream functional connectivity analyses. We counteracted this at four different stages. First, during tissue segmentation by careful isolation of the cerebellar tissue. Second, during fMRI data preprocessing, by using a one-step resampling (and thus interpolation) procedure. See also Supplementary Fig. 8 for the residual but negligible impact of this step on the volume, *R*_1_ and functional connectivity results. Third, during fMRI signal denoising, by regressing out WM and CSF signal timeseries at the voxel level. Finally, by modelling whole-brain functional connectivity as a graph during statistical analyses using the NBS, based on data from the entire study population.^58^ Together, these rendered the identified significant network minimally sensitive to cerebellar ROI- and/or patient-specific outliers.

## Conclusions

In summary, the current results indicate that the m.3243A > G mutation significantly impacts the cerebellum with the strongest changes observed in most severely affected patients, based on genetic, clinical and cognitive features. The impact of the m.3243A > G mutation ranges from reduced GM tissue integrity to impaired functional connectivity with cortical brain regions. Spatial characterization reveals that these changes occur especially in tissue and regions related to the FPN, crucial for information-processing speed and selective attention. Combined with our previous work,^16^ it provides insight into the neuropathological changes and a solid base to guide longitudinal studies aimed to track disease progression.

## Supplementary Material

fcac024_Supplementary_Data

## Abbreviations

BOLD =: blood-oxygen-level-dependent
DAN =: dorsal attention network
DMN =: default-mode network
eTIV =: estimated total intracranial volume
FPN =: frontoparietal network
FWE =: family-wise error
GM =: grey matter
iPSC =: induced pluripotent stem cell
NBS =: network-based statistics
NMDAS =: Newcastle Mitochondrial Disease Adult Scale
NPC =: non-parametric combination
LDST =: letter-digit substitution task
ROI =: region of interest
rs-fMRI =: resting-state functional MRI
SMN =: somatosensory motor network
VBM =: voxel-based morphometry
WM =: white matter
15-WLT =: 15 words-learning task

## References

[1] Taylor RW, Turnbull DM. Mitochondrial DNA mutations in human disease. Nat Rev Genet. 2005;6(5):389–402. 15861210 10.1038/nrg1606 PMC1762815

[2] Goto Y, Nonaka I, Horai S. A mutation in the tRNA(Leu)(UUR) gene associated with the MELAS subgroup of mitochondrial encephalomyopathies. Nature. 1990;348(6302):651–653. 2102678 10.1038/348651a0

[3] Manwaring N, Jones MM, Wang JJ, et al Population prevalence of the MELAS A3243G mutation. Mitochondrion. 2007;7(3):230–233. 17300999 10.1016/j.mito.2006.12.004

[4] Nesbitt V, Pitceathly RDS, Turnbull DM, et al The UK MRC mitochondrial disease patient cohort study: Clinical phenotypes associated with the m.3243A > G mutation—implications for diagnosis and management. J Neurol Neurosurg Psychiatry. 2013;84(8):936–938. 10.1136/jnnp-2012-303528 23355809

[5] de Laat P, Koene S, van den Heuvel LPWJ, Rodenburg RJT, Janssen MCH, Smeitink JAM. Clinical features and heteroplasmy in blood, urine and saliva in 34 Dutch families carrying the m.3243A > G mutation. J Inherit Metab Dis. 2012;35(6):1059–1069. 10.1007/s10545-012-9465-2 PMC3470685 22403016

[6] Hirano M, Ricci E, Koenigsberger MR, et al Melas: An original case and clinical criteria for diagnosis. Neuromuscul Disord. 1992;2(2):125–135. 1422200 10.1016/0960-8966(92)90045-8

[7] de Laat P, Rodenburg RR, Roeleveld N, Koene S, Smeitink JA, Janssen MC. Six-year prospective follow-up study in 151 carriers of the mitochondrial DNA 3243 A>G variant. J Med Genet. 2021;58(1):48–55. 32439810 10.1136/jmedgenet-2019-106800

[8] van den Ouweland JM, Lemkes HH, Ruitenbeek W, et al Mutation in mitochondrial tRNA(Leu)(UUR) gene in a large pedigree with maternally transmitted type II diabetes mellitus and deafness. Nat Genet. 1992;1(5):368–371. 1284550 10.1038/ng0892-368

[9] Lindroos MM, Borra RJ, Parkkola R, et al Cerebral oxygen and glucose metabolism in patients with mitochondrial m.3243A > G mutation. Brain. 2009;132(Pt 12):3274–3284. 19843652 10.1093/brain/awp259

[10] Tschampa HJ, Urbach H, Greschus S, Kunz WS, Kornblum C. Neuroimaging characteristics in mitochondrial encephalopathies associated with the m.3243A > G MTTL1 mutation. J Neurol. 2013;260(4):1071–1080. 23196335 10.1007/s00415-012-6763-4

[11] Tsujikawa K, Senda J, Yasui K, et al Distinctive distribution of brain volume reductions in MELAS and mitochondrial DNA A3243G mutation carriers: A voxel-based morphometric study. Mitochondrion. 2016;30:229–235. 27558483 10.1016/j.mito.2016.08.011

[12] Rodan LH, Poublanc J, Fisher JA, et al Cerebral hyperperfusion and decreased cerebrovascular reactivity correlate with neurologic disease severity in MELAS. Mitochondrion. 2015;22:66–74. 25801712 10.1016/j.mito.2015.03.002

[13] Kraya T, Neumann L, Paelecke-Habermann Y, et al Cognitive impairment, clinical severity and MRI changes in MELAS syndrome. Mitochondrion. 2019;44:53–57. 29289801 10.1016/j.mito.2017.12.012

[14] Bhatia KD, Krishnan P, Kortman H, Klostranec J, Krings T. Acute cortical lesions in MELAS syndrome: Anatomic distribution, symmetry, and evolution. Am J Neuroradiol. 2020;41(1):167–173. 31806591 10.3174/ajnr.A6325 PMC6975311

[15] Van den Ameele J, Hong Y, Manavaki R, et al [11C]PK11195-PET brain imaging of the mitochondrial translocator protein in mitochondrial disease. Neurology. 96(22) e2761–e2773. 10.1212/WNL.0000000000012033 PMC8205464 33883237

[16] Haast RAM, Ivanov D, IJsselstein RJT, et al Anatomic & metabolic brain markers of the m.3243A > G mutation: A multi-parametric 7 T MRI study. Neuroimage Clin. 2018;18:231–244. 29868447 10.1016/j.nicl.2018.01.017 PMC5984598

[17] Lax NZ, Hepplewhite PD, Reeve AK, et al Cerebellar ataxia in patients with mitochondrial DNA disease: A molecular clinicopathological study. J Neuropathol Exp Neurol. 2012;71(2):148–161. 22249460 10.1097/NEN.0b013e318244477d PMC3272439

[18] Picard M, McEwen BS. Mitochondria impact brain function and cognition. Proc Natl Acad Sci USA. 2014;111(1):7–8. 24367081 10.1073/pnas.1321881111 PMC3890847

[19] Sereno MI, Diedrichsen J, Tachrount M, Testa-Silva G, d’Arceuil H, De Zeeuw C. The human cerebellum has almost 80% of the surface area of the neocortex. Proc Natl Acad Sci USA. 2020;117(32):19538–19543. 32723827 10.1073/pnas.2002896117 PMC7431020

[20] Manto M, Bower JM, Conforto AB, et al Consensus paper: Roles of the cerebellum in motor control—The diversity of ideas on cerebellar involvement in movement. Cerebellum. 2012;11(2):457–487. 22161499 10.1007/s12311-011-0331-9 PMC4347949

[21] King M, Hernandez-Castillo CR, Poldrack RA, Ivry RB, Diedrichsen J. Functional boundaries in the human cerebellum revealed by a multi-domain task battery. Nat Neurosci. 2019;22(8):1371–1378. 31285616 10.1038/s41593-019-0436-x PMC8312478

[22] Buckner RL . The cerebellum and cognitive function: 25 years of insight from anatomy and neuroimaging. Neuron. 2013;80(3):807–815. 24183029 10.1016/j.neuron.2013.10.044

[23] Ramnani N . The primate cortico-cerebellar system: Anatomy and function. Nat Rev Neurosci. 2006;7(7):511–522. 16791141 10.1038/nrn1953

[24] Strick PL, Dum RP, Fiez JA. Cerebellum and nonmotor function. Annu Rev Neurosci. 2009;32:413–434. 19555291 10.1146/annurev.neuro.31.060407.125606

[25] Reeber SL, Otis TS, Sillitoe RV. New roles for the cerebellum in health and disease. Front Syst Neurosci. 2013;7:83. 24294192 10.3389/fnsys.2013.00083 PMC3827539

[26] Schaefer AM, Phoenix C, Elson JL, McFarland R, Chinnery PF, Turnbull DM. Mitochondrial disease in adults: A scale to monitor progression and treatment. Neurology. 2006;66(12):1932–1934. 16801664 10.1212/01.wnl.0000219759.72195.41

[27] Grady JP, Pickett SJ, Ng YS et al *.* mtDNA heteroplasmy level and copy number indicate disease burden in m.3243A > G mitochondrial disease. EMBO Mol Med. 2018;10(6):e8262. 29735722 10.15252/emmm.201708262 PMC5991564

[28] van der Elst W, van Boxtel MPJ, van Breukelen GJP, Jolles J. The letter digit substitution test: Normative data for 1,858 healthy participants aged 24–81 from the maastricht aging study (MAAS): Influence of age, education, and sex. J Clin Exp Neuropsychol. 2006;28(6):998–1009. 16822738 10.1080/13803390591004428

[29] Van der Elst W, Van Boxtel MPJ, Van Breukelen GJP, Jolles J. The Stroop color-word test: Influence of age, sex, and education; and normative data for a large sample across the adult age range. Assessment. 2006;13(1):62–79. 16443719 10.1177/1073191105283427

[30] Elst WVD, Boxtel MPJV, Breukelen GJPV, Jolles J. Rey’s verbal learning test: Normative data for 1855 healthy participants aged 24–81 years and the influence of age, sex, education, and mode of presentation. J Int Neuropsychol Soc. 2005;11(3):290–302. 15892905 10.1017/S1355617705050344

[31] Marques JP, Kober T, Krueger G, van der Zwaag W, Van de Moortele PF, Gruetter R. MP2RAGE, a self bias-field corrected sequence for improved segmentation and T1-mapping at high field. Neuroimage. 2010;49(2):1271–1281. 19819338 10.1016/j.neuroimage.2009.10.002

[32] Eggenschwiler F, Kober T, Magill AW, Gruetter R, Marques JP. SA2RAGE: A new sequence for fast B1+ -mapping. Magn Reson Med. 2012;67(6):1609–1619. 22135168 10.1002/mrm.23145

[33] Stüber C, Morawski M, Schäfer A, et al Myelin and iron concentration in the human brain: A quantitative study of MRI contrast. NeuroImage. 2014;93:95–106. 24607447 10.1016/j.neuroimage.2014.02.026

[34] Teeuwisse WM, Brink WM, Webb AG. Quantitative assessment of the effects of high-permittivity pads in 7 Tesla MRI of the brain. Magn Reson Med. 2012;67(5):1285–1293. 21826732 10.1002/mrm.23108

[35] Bazin PL, Weiss M, Dinse J, Schäfer A, Trampel R, Turner R. A computational framework for ultra-high resolution cortical segmentation at 7 Tesla. Neuroimage. 2014;93(Pt 2):201–209. 23623972 10.1016/j.neuroimage.2013.03.077

[36] Marques JP, Gruetter R. New developments and applications of the MP2RAGE sequence—focusing the contrast and high spatial resolution R1 mapping. PLoS One. 2013;8(7):e69294. 23874936 10.1371/journal.pone.0069294 PMC3712929

[37] Haast RAM, Ivanov D, Uludağ K. The impact of B1+ correction on MP2RAGE cortical T1 and apparent cortical thickness at 7T. Hum Brain Mapp. 2018;39(6):2412–2425. 29457319 10.1002/hbm.24011 PMC5969159

[38] Fischl B . FreeSurfer. Neuroimage. 2012;62(2):774–781. 22248573 10.1016/j.neuroimage.2012.01.021 PMC3685476

[39] Glasser MF, Sotiropoulos SN, Wilson JA, et al The minimal preprocessing pipelines for the human connectome project. Neuroimage. 2013;80:105–124. 23668970 10.1016/j.neuroimage.2013.04.127 PMC3720813

[40] Diedrichsen J . A spatially unbiased atlas template of the human cerebellum. Neuroimage. 2006;33(1):127–138. 16904911 10.1016/j.neuroimage.2006.05.056

[41] Ashburner J, Friston KJ. Voxel-based morphometry—the methods. Neuroimage. 2000;11(6 Pt 1):805–821. 10860804 10.1006/nimg.2000.0582

[42] Romero JE, Coupé P, Giraud R, et al CERES: A new cerebellum lobule segmentation method. Neuroimage. 2017;147:916–924. 27833012 10.1016/j.neuroimage.2016.11.003

[43] Yushkevich PA, Piven J, Hazlett HC, et al User-guided 3D active contour segmentation of anatomical structures: Significantly improved efficiency and reliability. Neuroimage. 2006;31(3):1116–1128. 16545965 10.1016/j.neuroimage.2006.01.015

[44] Ashburner J . A fast diffeomorphic image registration algorithm. Neuroimage. 2007;38(1):95–113. 17761438 10.1016/j.neuroimage.2007.07.007

[45] Diedrichsen J, Balsters JH, Flavell J, Cussans E, Ramnani N. A probabilistic MR atlas of the human cerebellum. Neuroimage. 2009;46(1):39–46. 19457380 10.1016/j.neuroimage.2009.01.045

[46] Good CD, Johnsrude IS, Ashburner J, Henson RN, Friston KJ, Frackowiak RS. A voxel-based morphometric study of ageing in 465 normal adult human brains. Neuroimage. 2001;14(1 Pt 1):21–36. 11525331 10.1006/nimg.2001.0786

[47] Cox RW . AFNI: Software for analysis and visualization of functional magnetic resonance neuroimages. Comput Biomed Res. 1996;29(3):162–173. 8812068 10.1006/cbmr.1996.0014

[48] Jenkinson M, Bannister P, Brady M, Smith S. Improved optimization for the robust and accurate linear registration and motion correction of brain images. Neuroimage. 2002;17(2):825–841. 12377157 10.1016/s1053-8119(02)91132-8

[49] Andersson JLR, Skare S, Ashburner J. How to correct susceptibility distortions in spin-echo echo-planar images: Application to diffusion tensor imaging. Neuroimage. 2003;20(2):870–888. 14568458 10.1016/S1053-8119(03)00336-7

[50] Greve DN, Fischl B. Accurate and robust brain image alignment using boundary-based registration. NeuroImage. 2009;48(1):63–72. 19573611 10.1016/j.neuroimage.2009.06.060 PMC2733527

[51] Jenkinson M, Beckmann CF, Behrens TEJ, Woolrich MW, Smith SM. FSL. Neuroimage. 2012;62(2):782–790. 21979382 10.1016/j.neuroimage.2011.09.015

[52] Whitfield-Gabrieli S, Nieto-Castanon A. *Conn*: A functional connectivity toolbox for correlated and anticorrelated brain networks. Brain Connect. 2012;2(3):125–141. 22642651 10.1089/brain.2012.0073

[53] Behzadi Y, Restom K, Liau J, Liu TT. A component based noise correction method (CompCor) for BOLD and perfusion based fMRI. Neuroimage. 2007;37(1):90–101. 17560126 10.1016/j.neuroimage.2007.04.042 PMC2214855

[54] Marcus DS, Harwell J, Olsen T, et al Informatics and data mining tools and strategies for the human connectome project. Front Neuroinform. 2011;5:4. 21743807 10.3389/fninf.2011.00004 PMC3127103

[55] Schaefer A, Kong R, Gordon EM, et al Local-global parcellation of the human cerebral cortex from intrinsic functional connectivity MRI. Cereb Cortex. 2018;28(9):3095–3114. 28981612 10.1093/cercor/bhx179 PMC6095216

[56] Yeo BTT, Krienen FM, Sepulcre J, et al The organization of the human cerebral cortex estimated by intrinsic functional connectivity. J Neurophysiol. 2011;106(3):1125–1165. 21653723 10.1152/jn.00338.2011 PMC3174820

[57] Winkler AM, Ridgway GR, Webster MA, Smith SM, Nichols TE. Permutation inference for the general linear model. Neuroimage. 2014;92:381–397. 24530839 10.1016/j.neuroimage.2014.01.060 PMC4010955

[58] Zalesky A, Fornito A, Bullmore ET. Network-based statistic: Identifying differences in brain networks. Neuroimage. 2010;53(4):1197–1207. 20600983 10.1016/j.neuroimage.2010.06.041

[59] Winkler AM, Webster MA, Brooks JC, Tracey I, Smith SM, Nichols TE. Non-parametric combination and related permutation tests for neuroimaging. Hum Brain Mapp. 2016;37(4):1486–1511. 26848101 10.1002/hbm.23115 PMC4783210

[60] Alberton BAV, Nichols TE, Gamba HR, Winkler AM. Multiple testing correction over contrasts for brain imaging. Neuroimage. 2020;216:116760. 32201328 10.1016/j.neuroimage.2020.116760 PMC8191638

[61] Rubin TN, Koyejo O, Gorgolewski KJ, Jones MN, Poldrack RA, Yarkoni T. Decoding brain activity using a large-scale probabilistic functional-anatomical atlas of human cognition. PLoS Comput Biol. 2017;13(10):e1005649. 29059185 10.1371/journal.pcbi.1005649 PMC5683652

[62] Yarkoni T, Poldrack RA, Nichols TE, Van Essen DC, Wager TD. Large-scale automated synthesis of human functional neuroimaging data. Nat Methods. 2011;8(8):665–670. 21706013 10.1038/nmeth.1635 PMC3146590

[63] Köster J, Rahmann S. Snakemake—a scalable bioinformatics workflow engine. Bioinformatics. 2012;28(19):2520–2522. 22908215 10.1093/bioinformatics/bts480

[64] Herron TJ, Kang X, Woods DL. Sex differences in cortical and subcortical human brain anatomy. F1000Res. 2015;4:88.

[65] Magistretti PJ, Pellerin L, Rothman DL, Shulman RG. Energy on demand. Science. 1999;283(5401):496–497. 9988650 10.1126/science.283.5401.496

[66] Krebs HA, Johnson WA. Metabolism of ketonic acids in animal tissues. Biochem J. 1937;31(4):645–660. 16746382 10.1042/bj0310645 PMC1266984

[67] Russell O, Turnbull D. Mitochondrial DNA disease-molecular insights and potential routes to a cure. Exp Cell Res. 2014;325(1):38–43. 24675282 10.1016/j.yexcr.2014.03.012 PMC4058519

[68] El-Hattab AW, Adesina AM, Jones J, Scaglia F. MELAS syndrome: Clinical manifestations, pathogenesis, and treatment options. Mol Genet Metab. 2015;116(1–2):4–12. 26095523 10.1016/j.ymgme.2015.06.004

[69] Sasarman F, Antonicka H, Shoubridge EA. The A3243G tRNALeu(UUR) MELAS mutation causes amino acid misincorporation and a combined respiratory chain assembly defect partially suppressed by overexpression of EFTu and EFG2. Hum Mol Genet. 2008;17(23):3697–3707. 18753147 10.1093/hmg/ddn265

[70] Klein Gunnewiek TM, Van Hugte EJH, Frega M, et al m.3243A > G-induced mitochondrial dysfunction impairs human neuronal development and reduces neuronal network activity and synchronicity. Cell Rep. 2020;31(3):107538. 32320658 10.1016/j.celrep.2020.107538

[71] Quintana A, Kruse SE, Kapur RP, Sanz E, Palmiter RD. Complex I deficiency due to loss of Ndufs4 in the brain results in progressive encephalopathy resembling Leigh syndrome. Proc Natl Acad Sci USA. 2010;107(24):10996–11001. 20534480 10.1073/pnas.1006214107 PMC2890717

[72] Aryaman J, Johnston IG, Jones NS. Mitochondrial DNA density homeostasis accounts for a threshold effect in a cybrid model of a human mitochondrial disease. Biochem J. 2017;474(23):4019–4034. 29079678 10.1042/BCJ20170651 PMC5705840

[73] Rossignol R, Faustin B, Rocher C, Malgat M, Mazat JP, Letellier T. Mitochondrial threshold effects. Biochem J. 2003;370(Pt 3):751–762. 12467494 10.1042/BJ20021594 PMC1223225

[74] Apps R, Hawkes R. Cerebellar cortical organization: A one-map hypothesis. Nat Rev Neurosci. 2009;10(9):670–681. 19693030 10.1038/nrn2698

[75] Apps R, Garwicz M. Anatomical and physiological foundations of cerebellar information processing. Nat Rev Neurosci. 2005;6(4):297–311. 15803161 10.1038/nrn1646

[76] Brochu G, Maler L, Hawkes R. Zebrin II: A polypeptide antigen expressed selectively by Purkinje cells reveals compartments in rat and fish cerebellum. J Comp Neurol. 1990;291(4):538–552. 2329190 10.1002/cne.902910405

[77] Ahn AH, Dziennis S, Hawkes R, Herrup K. The cloning of zebrin II reveals its identity with aldolase C. Development. 1994;120(8):2081–2090. 7925012 10.1242/dev.120.8.2081

[78] Arakaki TL, Pezza JA, Cronin MA, et al Structure of human brain fructose 1,6-(bis)phosphate aldolase: Linking isozyme structure with function. Protein Sci. 2004;13(12):3077–3084. 15537755 10.1110/ps.04915904 PMC2287316

[79] Gallo G . The bioenergetics of neuronal morphogenesis and regeneration: Frontiers beyond the mitochondrion. Dev Neurobiol. 2020;80(7–8):263–276. 32750228 10.1002/dneu.22776 PMC7749811

[80] Hernandez-Castillo CR, King M, Diedrichsen J, Fernandez-Ruiz J. Unique degeneration signatures in the cerebellar cortex for spinocerebellar ataxias 2, 3, and 7. NeuroImage: Clin. 2018;20:931–938. 30308379 10.1016/j.nicl.2018.09.026 PMC6178193

[81] Kelly RM, Strick PL. Cerebellar loops with motor cortex and prefrontal cortex of a nonhuman primate. J Neurosci. 2003;23(23):8432–8444. 12968006 10.1523/JNEUROSCI.23-23-08432.2003 PMC6740694

[82] Ramnani N . Frontal lobe and posterior parietal contributions to the cortico-cerebellar system. Cerebellum. 2012;11(2):366–383. 21671065 10.1007/s12311-011-0272-3

[83] Biswal B, Yetkin FZ, Haughton VM, Hyde JS. Functional connectivity in the motor cortex of resting human brain using echo-planar MRI. Magn Reson Med. 1995;34(4):537–541. 8524021 10.1002/mrm.1910340409

[84] Buckner RL, Krienen FM, Castellanos A, Diaz JC, Yeo BTT. The organization of the human cerebellum estimated by intrinsic functional connectivity. J Neurophysiol. 2011;106(5):2322–2345. 21795627 10.1152/jn.00339.2011 PMC3214121

[85] Sokolov AA, Miall RC, Ivry RB. The cerebellum: Adaptive prediction for movement and cognition. Trends Cogn Sci. 2017;21(5):313–332. 28385461 10.1016/j.tics.2017.02.005 PMC5477675

[86] Moberget T, Doan NT, Alnæs D, et al Cerebellar volume and cerebellocerebral structural covariance in schizophrenia: A multisite mega-analysis of 983 patients and 1349 healthy controls. Mol Psychiatry. 2018;23(6):1512–1520. 28507318 10.1038/mp.2017.106

[87] Menon V . Large-scale brain networks and psychopathology: A unifying triple network model. Trends Cogn Sci. 2011;15(10):483–506. 21908230 10.1016/j.tics.2011.08.003

[88] Marek S, Siegel JS, Gordon EM, et al Spatial and temporal organization of the individual human cerebellum. Neuron. 2018;100(4):977–993.e7. 30473014 10.1016/j.neuron.2018.10.010 PMC6351081

[89] Schmahmann JD . Disorders of the cerebellum: Ataxia, dysmetria of thought, and the cerebellar cognitive affective syndrome. J Neuropsychiatry Clin Neurosci. 2004;16(3):367–378. 15377747 10.1176/jnp.16.3.367

[90] Schmahmann JD, Sherman JC. The cerebellar cognitive affective syndrome. Brain. 1998;121(Pt 4):561–579. 9577385 10.1093/brain/121.4.561

[91] Moore HL, Kelly T, Bright A, et al Cognitive deficits in adult m.3243A > G- and m.8344A > G-related mitochondrial disease: Importance of correcting for baseline intellectual ability. Ann Clin Transl Neurol. 2019;6(5):826–836. 31139680 10.1002/acn3.736 PMC6529924

[92] Bundesen C, Habekost T, Kyllingsbaek S. A neural theory of visual attention: Bridging cognition and neurophysiology. Psychol Rev. 2005;112(2):291–328. 15783288 10.1037/0033-295X.112.2.291

[93] Küchenhoff S, Sorg C, Schneider SC, et al Visual processing speed is linked to functional connectivity between right frontoparietal and visual networks. Eur J Neurosci. 2021;53(10):3362–3377. 33764572 10.1111/ejn.15206

[94] Wang R, Lin J, Sun C, et al Topological reorganization of brain functional networks in patients with mitochondrial encephalomyopathy with lactic acidosis and stroke-like episodes. Neuroimage Clin. 2020;28:102480. 33395972 10.1016/j.nicl.2020.102480 PMC7645289

[95] Desmond JE, Gabrieli JD, Glover GH. Dissociation of frontal and cerebellar activity in a cognitive task: Evidence for a distinction between selection and search. Neuroimage. 1998;7(4 Pt 1):368–376. 9626676 10.1006/nimg.1998.0340

[96] Stuss DT, Floden D, Alexander MP, Levine B, Katz D. Stroop performance in focal lesion patients: Dissociation of processes and frontal lobe lesion location. Neuropsychologia. 2001;39(8):771–786. 11369401 10.1016/s0028-3932(01)00013-6

[97] Pujol J, Vendrell P, Deus J, et al The effect of medial frontal and posterior parietal demyelinating lesions on stroop interference. Neuroimage. 2001;13(1):68–75. 11133310 10.1006/nimg.2000.0662

[98] Usui N, Haji T, Maruyama M, et al Cortical areas related to performance of WAIS Digit Symbol Test: A functional imaging study. Neurosci Lett. 2009;463(1):1–5. 19631255 10.1016/j.neulet.2009.07.048

[99] Hickok G, Poeppel D. Dorsal and ventral streams: A framework for understanding aspects of the functional anatomy of language. Cognition. 2004;92(1–2):67–99. 15037127 10.1016/j.cognition.2003.10.011

[100] Rodan LH, Poublanc J, Fisher JA, Sobczyk O, Mikulis DJ, Tein I. L-arginine effects on cerebrovascular reactivity, perfusion and neurovascular coupling in MELAS (mitochondrial encephalomyopathy with lactic acidosis and stroke-like episodes) syndrome. PLoS One. 2020;15(9):e0238224. 32881886 10.1371/journal.pone.0238224 PMC7470264

